# Exercise as an anti-inflammatory Therapy in Axial Spondyloarthritis Therapeutic Intervention (EXTASI) study: a randomized controlled trial

**DOI:** 10.1093/rap/rkae062

**Published:** 2024-05-11

**Authors:** Matthew J Roberts, Malik Hamrouni, Victoria Linsley, Arumugam Moorthy, Nicolette C Bishop

**Affiliations:** National Centre for Sport and Exercise Medicine, School of Sport, Exercise and Health Sciences, Loughborough University, Loughborough, UK; National Institute for Health Research Leicester Biomedical Research Centre, University Hospitals of Leicester, National Health Service Trust and the University of Leicester, Leicester, UK; National Centre for Sport and Exercise Medicine, School of Sport, Exercise and Health Sciences, Loughborough University, Loughborough, UK; National Institute for Health Research Leicester Biomedical Research Centre, University Hospitals of Leicester, National Health Service Trust and the University of Leicester, Leicester, UK; National Centre for Sport and Exercise Medicine, School of Sport, Exercise and Health Sciences, Loughborough University, Loughborough, UK; National Institute for Health Research Leicester Biomedical Research Centre, University Hospitals of Leicester, National Health Service Trust and the University of Leicester, Leicester, UK; National Centre for Sport and Exercise Medicine, School of Sport, Exercise and Health Sciences, Loughborough University, Loughborough, UK; Department of Rheumatology, University Hospitals of Leicester NHS Trust, Leicester, UK; National Centre for Sport and Exercise Medicine, School of Sport, Exercise and Health Sciences, Loughborough University, Loughborough, UK; National Institute for Health Research Leicester Biomedical Research Centre, University Hospitals of Leicester, National Health Service Trust and the University of Leicester, Leicester, UK

**Keywords:** axial spondyloarthritis, exercise, spinal pain, inflammation, cardiovascular disease

## Abstract

**Objectives:**

Axial SpA (axSpA) is a chronic inflammatory disease, yet despite known anti-inflammatory effects of exercise, the effect of exercise on inflammatory immune cell populations and associated inflammatory profiles in axSpA is unknown. This randomized controlled trial investigated the effect of 12 weeks of walking on symptom severity, cardiometabolic health, inflammatory biomarkers and immune cell populations.

**Methods:**

Twenty people (60% male) living with axSpA who were on a stable dose of NSAIDs participated. Participants were randomly assigned to control or exercise (30 min of walking five times per week). Participants were invited back every 4 weeks for assessment.

**Results:**

There was a 0% dropout rate and no adverse events in the exercise group, showing walking exercise was well tolerated. Home-based walking for 12 weeks lowered the proportion of pro-inflammatory monocytes, whereas they increased in the control group. Changes were associated with lower IL-6 and CRP concentrations, lower spinal pain and lower systolic blood pressure in the exercise group, whereas these markers increased in the control group. Reductions in IL-6 and pro-inflammatory monocytes with exercise were independent of lower body fat percentage.

**Conclusions:**

Supplementing NSAID therapy with walking exercise can improve inflammatory immune profiles in people with axSpA, coinciding with reductions in spinal pain. Importantly, the exercise was well tolerated, suggesting walking exercise can be used as an adjuvant anti-inflammatory therapy for NSAID treatments. This should now be explored in people living with axSpA who have had high enough disease activity to necessitate the prescription of biologic or synthetic DMARD treatments.

**Trial registration:**

ClinicalTrials.gov (http://clinicaltrials.gov), NCT04368494.

## Graphical Abstract

**Figure:**
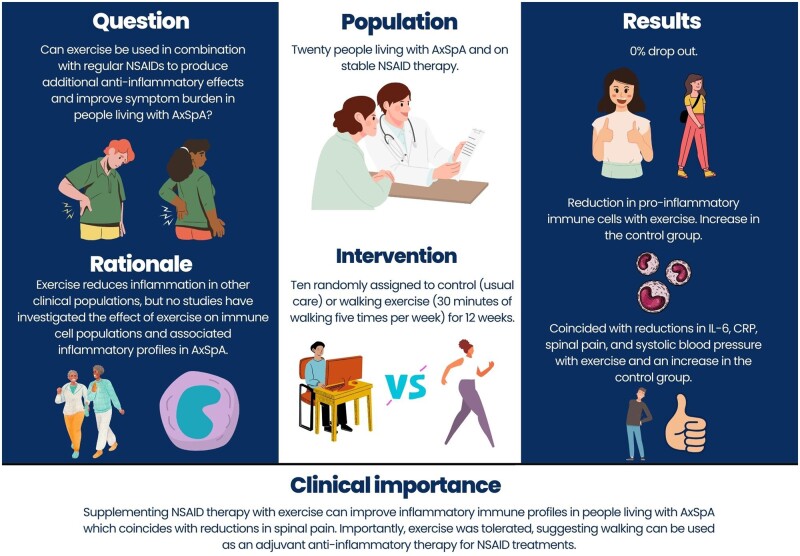

Key messages

- Walking can optimize NSAID therapy to enhance additional anti-inflammatory benefits in axSpA management.
- Walking can augment NSAID therapy to reduce spinal pain and fatiguability.
- Exercise in the form of walking is well-tolerated by patients with axSpA without cardiovascular disease.

## Introduction

Axial SpA (axSpA) is a chronic inflammatory disease affecting people at a young age, with a prevalence of 0.8% and a median delay in diagnosis of 2–6 years [1]. The main symptoms include inflammatory back pain, fatigue and disability [2]. However, axSpA also significantly impacts quality of life and mental health, with anxiety and depression being 6-fold higher in people living with axSpA compared with the general population [3].

Increasing evidence highlights an elevated risk of cardiovascular disease (CVD) with axSpA, mediated by greater systemic inflammation [4]. Concentrations of inflammatory markers, including TNF-α, CRP, IL-6 and IL-17, are elevated with axSpA and strongly correlate with CVD events [4]. Accordingly, a recent meta-analysis (2021) reported myocardial infarction, stroke and all-cause mortality to be 52%, 21% and 23% higher, respectively, in people with axSpA compared with the general population [5].

NSAIDs are used as an initial treatment strategy for axSpA [6]. When ineffective, people with axSpA are progressed to biologic medications, which target receptors for specific pro-inflammatory cytokines (mainly IL-17) that are expressed on many cells, including monocytes [6]. Biologics aim to prevent inflammatory cytokine and chemokine release, reducing local and systemic inflammation to improve disease outcomes [7]. However, biologic therapy is relatively expensive and outcomes are influenced by disease duration, baseline inflammation, age and functional impairment [8].

Exercise can lower the risk of developing inflammatory diseases in the general population due to improvements in cardiometabolic health [9] and the anti-inflammatory effects of exercise [10]. Consequently, exercise may be an integral part in the management of axSpA. In clinical populations, exercise has been shown to lower the proportion of intermediate and non-classical monocytes [11] that express the cell surface marker CD16 [12]. CD16^+^ monocytes are a major source of inflammatory cytokines and are elevated with inflammatory diseases, including CVD [13]. In people living with axSpA, exercise programs have produced moderate improvements in subjective disease activity, function and spinal mobility [14]. However, few studies have investigated the anti-inflammatory effects of exercise in people living with axSpA, and normally these are limited to the analysis of CRP concentrations [8]. CRP is highly variable and can significantly change on a day-to-day basis, suggesting that it is not the most reliable marker of inflammation [8]. Also, CRP is elevated in only ≈40% of those living with axSpA and does not correlate with disease activity in >50% of those living with axSpA [15].

Given the importance of inflammation with axSpA, the present randomized controlled trial investigated the effect of 12 weeks of walking on subjective measures of symptom severity, cardiometabolic blood markers, inflammatory immune cell populations and plasma biomarkers of inflammation in people with axSpA and regularly taking NSAIDs.

We hypothesized that the exercise group would have improvements in self-reported outcomes such as disease activity and spinal pain compared with the control group, which would be supported by reductions in CD16^+^ monocytes and concentrations of inflammatory markers in the blood.

## Methods

### Trial design

The trial was a randomized, controlled, observer-blinded, superiority trial with two parallel groups with a 1:1 ratio, approved by the West Midlands Research Ethics Committee (Solihull; 19/WM/0308) and registered at ClinicalTrials.gov (NCT04368494). The original protocol included a follow-up data collection point at week 20. This was removed before trial commencement to reduce participant burden.

### Participants

Patients with medically diagnosed axSpA across the University Hospitals of Leicester (UHL) Trust were telephoned by the consultant rheumatologist to discuss the study and screen for eligibility criteria, which included receiving NSAID treatment, no history of heart disease or anaemia, not pregnant or planning pregnancy and able to commit to the time and walking demands of the study (participants reporting that they cannot physically walk were excluded). A history of heart disease was an exclusion criterion, as an exercise ECG was not available. Participants were not approached based on ethnicity, but the UHL Trust has a relatively high percentage of South Asians [16]. Potential participants were invited to the clinic to discuss the research study in more detail with the lead researcher and participants left with an information pack that included contact details for the researcher. The potential participants were then given at least 7 days to decide whether to participate and e-mailed the lead researcher to express interest.

Interested participants were invited back to the clinic to provide written informed consent and complete an international physical activity questionnaire (IPAQ) to exclude participants who scored high (≥1 h of moderate intensity activity per day), as this would interfere with the exercise intervention [17]. Participants were fitted with an accelerometer (ActiGraph GT3x, Actigraph, Pensacola, FL, USA) to quantify habitual physical activity across 7 days before the first laboratory visit. Time spent in light, moderate or vigorous activity was determined using counts per minute [18]. Subsequent visits and data collection were conducted at Loughborough University.

### Interventions

Participants maintained their diet for 7 days and avoided strenuous physical activity and caffeine for 48 h before their first laboratory visit. Participants arrived in a fasted state (>10 h fast). Questionnaires to assess symptom severity were completed. Anthropometrics were taken, then participants lay down in a semi-supine position for 5 min for a blood pressure (BP) measurement. Ethylenediaminetetraacetic acid (EDTA) and sodium heparin blood samples were collected (BD Valu-Set, Becton-Dickinson, Stockholm, Sweden) before participants ate a small snack in preparation for the physical function tests. Supervised five times sit-to-stand (STS5) and 60-s sit-to-stand (STS60) tests assessed muscular strength and endurance [19, 20]. Participants were fitted with an activity band (Mi Smart Band 5, Xiomi, Beijing, China) with heart rate functionality then walked for 30 min at a pace of ‘somewhat hard’ based on their rating of perceived exertion [21].

Participants were invited into a separate room with the principal investigator where they were told if they had been assigned to the usual care (control) or exercise intervention. Participants in the control group returned their activity band and were asked to maintain their normal behaviour. The exercise group kept their activity band and were asked to complete 30 min of walking, five times per week, at a pace of somewhat hard. An exercise diary to record the date of exercise, duration of exercise, heart rate and rating of perceived exertion was provided. Participants were invited back every 4 weeks for 12 weeks. Participants were contacted every 2 weeks by the principal investigator to schedule visits and answer any concerns with regard to walking in the exercise group.

#### Outcome measures

**Figure 1.**
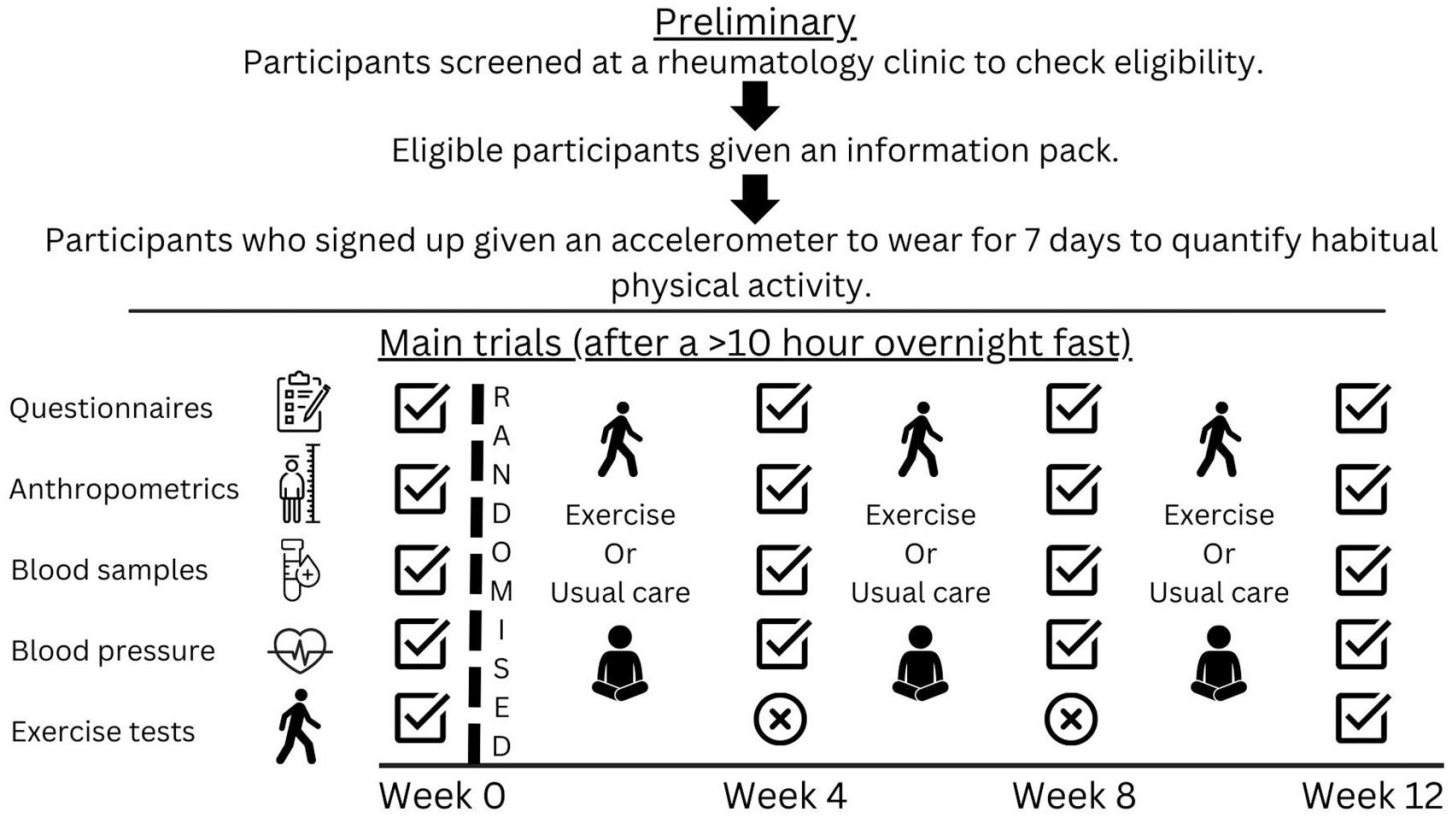
Study protocol. Participants were randomly assigned to the control (usual care) or exercise (30 min of walking five times per week) at the end of the baseline visit (week 0). Participants were then invited back every 4 weeks to complete questionnaires, anthropometrics, blood samples and BP to evaluate the effectiveness of the intervention. Physical function and cardiorespiratory tests were conducted at weeks 0 and 12

Questionnaires, anthropometrics, blood samples and BP were completed at all visits. Physical function tests were only completed at weeks 0 and 12 (Fig. 1). Disease activity (BASDAI), physical function (BASFI), work productivity [Work Productivity and Activity Impairment (WPAI)], health index [Assessment of SpondyloArthritis international Society Health Index (ASAS-HI)] and spinal pain (visual analogue scale of 1–10) were primary outcomes and assessed via questionnaire. Monocyte phenotypes were primary outcomes and assessed at all visits. Gating strategies for assessment of monocyte phenotypes are shown in Supplementary Fig. S1, available at *Rheumatology Advances in Practice* online [22]. A total of 120 µL of sodium heparin whole blood was incubated with CD14-FITC and CD16-PE for 10 min in the dark before being lysed (Becton Dickinson, Oxford, UK) for 10 min in the dark. Samples were then centrifuged at 3500 *g* for 6 min at 4°C. Residue was aspirated and FACS buffer added before being centrifuged as above. Residue was aspirated again and cells were resuspended in 300 µL of PBS (Thermo Fisher Scientific, Loughborough, UK). Unstained and single-stained controls were prepared as above to establish gates and compensation on the flow cytometer (BD Accuri C6, Becton Dickinson, Oxford, UK).

EDTA plasma concentrations of triacylglycerol, total cholesterol, high-density lipoprotein cholesterol, low-density lipoprotein cholesterol, glucose and CRP concentrations were secondary outcomes and assessed at all visits using commercially available kits and a benchtop analyser (Pentra 400, Horiba Medical, Montpelier, France). Plasma IL-6 was determined using ELISAs (R&D Systems, Abingdon, UK). Anthropometrics and BP were secondary outcomes and assessed at all visits. Stature and body mass were measured using a fixed wall stadiometer and scale (Seca, Hamburg, Germany). Waist and hip circumference were measured using a non-elastic measuring tape (Hokanson, Bellevue, WA, USA) [23]. Body fat percentage was estimated using biological impedance analysis (mBCA 515, Seca, Hamburg, Germany). STS5 and STS60 tests were secondary outcomes and assessed at weeks 0 and 12.

### Sample size

An arbitrary sample size of 20 was selected due to a lack of existing monocyte data in people with axSpA and this is the first study of this hypothesis.

### Randomization

Participants were randomly assigned to either the usual care (control, *n* = 10) or exercise group (*n* = 10) via a random number generator. This was performed by the principal investigator, who communicated trial allocation to participants at the end of the week 0 visit. The lead researcher who was responsible for data collection and analysis was blinded to trial allocation for the duration of the study.

### Statistical analysis

Data were analysed using the SPSS version 27 (IBM, Armonk, NY, USA). The Shapiro–Wilk test and histograms explored normality. Outliers were identified as ±2.5 s.d. around the mean for normally distributed data and the median for skewed data. Skewed data were natural log transformed prior to analysis and analysis was performed on the log-arithmetic transformation of the data, but then back-transformed for presentation [24]. Data are presented as mean (s.d.) unless stated otherwise. Statistical significance was accepted as *P* < 0.05. Effect sizes (ESs) are presented to supplement findings. An ES of 0.2 was considered as the minimum value for a meaningful difference [25]. ESs of ≥0.5 and ≥0.8 were considered medium and large, respectively [25].

Independent *t*-tests assessed baseline differences between groups (exercise *vs* control). General linear models (GLMs) assessed group × time effects pre- and post-intervention on outcomes between groups. We then conducted 2 × 4 mixed ANOVA on the key outcomes—subjective measures of symptom severity, concentrations of cardiovascular and inflammatory markers and proportions of monocyte subsets—to assess whether any change in an outcome was the result of an interaction between group and time; e.g. if improvements were seen within the first 4 weeks rather than over the 12 weeks.

### Sensitivity analyses

Sensitivity analyses were performed for CRP due to an outlier at baseline in the exercise group. The data for *n* = 1 was removed and statistical analyses performed again. Sensitivity analyses were also performed for outcomes with significant baseline differences between the groups. Adjustment was performed by repeating the above statistical procedures with baseline measurements as a covariate. Controlling for reductions in body fat percentage by including it as a covariate for IL-6 and non-classical monocytes was performed to assess whether reductions in body fat contributed to lower IL-6 and non-classical monocytes at follow-up.

## Results

### Participant flow

**Figure 2.**
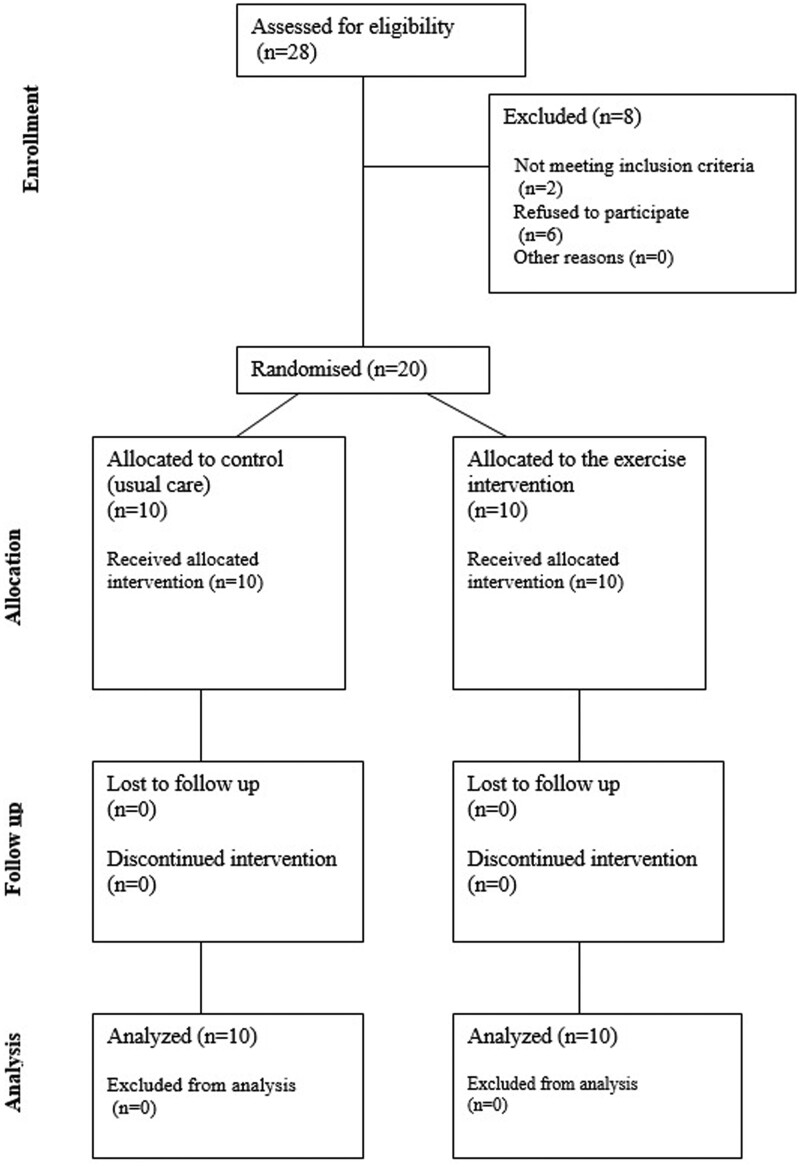
Consolidated Standards of Reporting Trials diagram

A total of 28 participants were initially screened for eligibility and 8 participants did not fit the criteria (Fig. 2). These 20 participants (60% male, 70% Caucasian, 30% South Asian) provided written informed consent. There was a 0% dropout rate across the 12 weeks and no flare-ups in the exercise group, showing walking exercise was well tolerated (Fig. 2). All visits happened between January and September 2022.

### Baseline group differences

There were no substantial differences in participant characteristics between groups at baseline (Table 1).

**Table 1.** Participant characteristics at baseline

Variables	Control (*n *=* *10)	Exercise (*n *=* *10)
Age (years), mean (s.d.)	43.6 (10.1)	47.1 (11.4)
Male, %	70	50
Ethnicity, %		
White British	70	70
South Asian	30	30
Disease duration (years), mean (s.d.)	8.6 (5.0)	8.3 (5.7)
Stature (cm), mean (s.d.)	170.9 (6.4)	173.7 (8.9)
Body mass (kg), mean (s.d.)	79.4 (15.9)	90.1 (20.9)
Low physical activity (min/day), mean (s.d.)	557 (80)	538 (31)
Light physical activity (min/day), mean (s.d.)	347 (23)	356 (25)
Moderate–vigorous physical activity (min/day), mean (s.d.)	17.8 (3.5)	18.3 (9.7)
Daily steps, mean (s.d.)	6348 (1458)	6691 (2790)

Data were analysed using independent *t*-tests (exercise *vs* control). Disease duration is the number of years since being clinically diagnosed.

### Effect of intervention

A significant interaction effect for waist circumference, body fat percentage and systolic BP was detected (Table 2;*P* ≤ 0.044). Waist circumference decreased by 1.8 cm (95% CI −2.8, −0.7) in the exercise group but increased by 0.4 cm (95% CI −0.7, 1.5) in the control group. Body fat percentage decreased by 2.5% (95% CI −3.9, −1.1) in the exercise group but increased by 0.4% (95% CI −0.9, 1.7) in the control group. Systolic BP decreased by 3 mmHg (95% CI −7, 1) in the exercise group but increased by 3 mmHg (95% CI −1, 7) in the control group.

**Table 2.** Physical, cardiovascular and subjective disease parameters at baseline and after 12 weeks between the control (*n* = 10) and exercise (*n* = 10) groups

Variable	Control group		Exercise group		*P*-value (ES)	
	Baseline	12 weeks	Baseline	12 weeks	Baseline group differences^a^	12-week interaction effect^b^
Body mass index (kg/m^2^)	27.2 (5.0)	27.2 (5.1)	29.6 (5.0)	29.1 (4.9)	0.284 (0.49)	0.065
Waist circumference (cm)	93.0 (17.6)	93.4 (17.8)	95.4 (15.1)	93.6 (14.5)	0.747 (0.15)	**0.008**
Hip circumference (cm)	101.8 (9.8)	102.0 (10.3)	106.6 (7.8)	106.6 (8.5)	0.240 (0.54)	0.865
Waist:hip ratio	0.91 (0.12)	0.91 (0.13)	0.89 (0.10)	0.87 (0.10)	0.721 (0.16)	0.110
Body fat percentage (%)	32.1 (8.7)	32.5 (7.1)	37.6 (6.0)	35.4 (6.0)	0.099 (0.78)	**0.006**
Systolic blood pressure (mmHg)	131 (23)	134 (24)	129 (13)	126 (13)	0.851 (0.09)	**0.044**
Diastolic blood pressure (mmHg)	88 (10)	90 (13)	91 (9)	90 (8)	0.476 (0.33)	0.194
Sit-to-stand 5 test (seconds)	12.2 (5.3)	9.7 (3.3)	11.3 (4.7)	9.4 (5.0)	0.682 (0.19)	0.599
Sit-to-stand 60 test (number)	29 (10)	33 (12)	27 (7)	34 (11)	0.574 (0.26)	0.394
Total cholesterol (mmol/l)	4.09 (0.61)	3.99 (0.23)	4.74 (0.60)	4.52 (0.35)	**0.028 (1.07)**	0.464
HDL-C (mmol/l)	1.28 (0.23)	1.24 (0.14)	1.45 (0.35)	1.36 (0.33)	0.191 (0.61)	0.405
LDL-C (mmol/l)	2.85 (0.65)	2.83 (0.67)	3.17 (0.63)	3.03 (0.53)	0.284 (0.49)	0.480
TAG (mmol/l)	0.76 (0.28)	0.72 (0.32)	1.23 (0.68)	1.33 (0.82)	0.060 (0.90)	0.367
Glucose (mmol/l)	4.86 (0.43)	4.78 (0.44)	4.56 (0.39)	4.63 (0.36)	0.125 (0.72)	0.327
CRP (mg/l), median (IQR)	5.36 (1.99–8.73)	8.47 (5.30–11.64)	2.66 (0.00–6.03)	1.86 (0.00–5.02)	0.250 (0.53)	0.092
IL-6 (pg/ml)	4.66 (1.19)	4.76 (1.12)	3.55 (1.16)	2.82 (0.1.06)	0.062 (0.89)	**0.002**
White blood cells (10^9^/L)	6.38 (1.45)	6.27 (1.47)	6.12 (1.57)	6.26 (2.04)	0.702 (0.17)	0.561
Monocytes (10^9^/L)	0.47 (0.09)	0.46 (0.11)	0.47 (0.13)	0.49 (0.08)	0.889 (0.06)	0.606
Lymphocytes (10^9^/L)	1.66 (0.42)	1.60 (0.38)	1.99 (0.60)	1.86 (0.47)	0.167 (0.64)	0.675
Classical monocytes (%)	59.6 (11.1)	50.8 (7.5)	52.5 (8.2)	59.2 (8.1)	0.119 (0.73)	**<0.001**
Classical monocytes (cells/µL)	281 (73)	235 (100)	245 (52)	288 (45)	0.224 (0.56)	**0.006**
Intermediate monocytes (%)	5.8 (3.4)	5.3 (1.9)	4.5 (1.5)	5.0 (1.2)	0.225 (0.53)	0.395
Intermediate monocytes (cells/µL)	29 (20)	26 (17)	22 (9)	25 (10)	0.344 (0.43)	0.428
Non-classical monocytes (%)	34.4 (9.7)	43.9 (7.0)	42.9 (7.2)	35.8 (8.0)	**0.039 (1.00)**	**<0.001**
Non-classical monocytes (cells/µL)	161 (47)	200 (48)	210 (83)	177 (55)	0.119 (0.73)	**<0.001**
BASDAI	3.0 (1.4)	3.5 (1.8)	4.0 (1.9)	3.7 (2.4)	0.201 (0.31)	0.244
BASFI	2.5 (1.6)	2.5 (1.5)	2.7 (1.9)	2.6 (2.5)	0.744 (0.29)	0.694
Spinal pain	1.9 (1.2)	3.1 (2.5)	2.7 (2.5)	1.6 (1.8)	0.355 (0.49)	**0.049**
WPAI	4.7 (2.9)	7.7 (4.0)	4.7 (5.1)	4.6 (5.4)	1.000 (0.05)	0.063
ASAS-HI	6.3 (3.8)	4.3 (3.3)	7.3 (3.4)	6.8 (4.2)	0.541 (0.36)	0.333

Data are presented as mean (s.d.) unless stated otherwise. Significant values are in bold.

Data were analysed using generalised linear models with group (exercise *vs* control) and group × time interactions modelled as fixed factors.

a Baseline comparison between groups (*P* ≤ 0.039).

b Group × time interaction effect (*P* ≤ 0.049).

LDL-C: low-density lipoprotein cholesterol; TAG: triacylglycerol.

**Figure 3.**
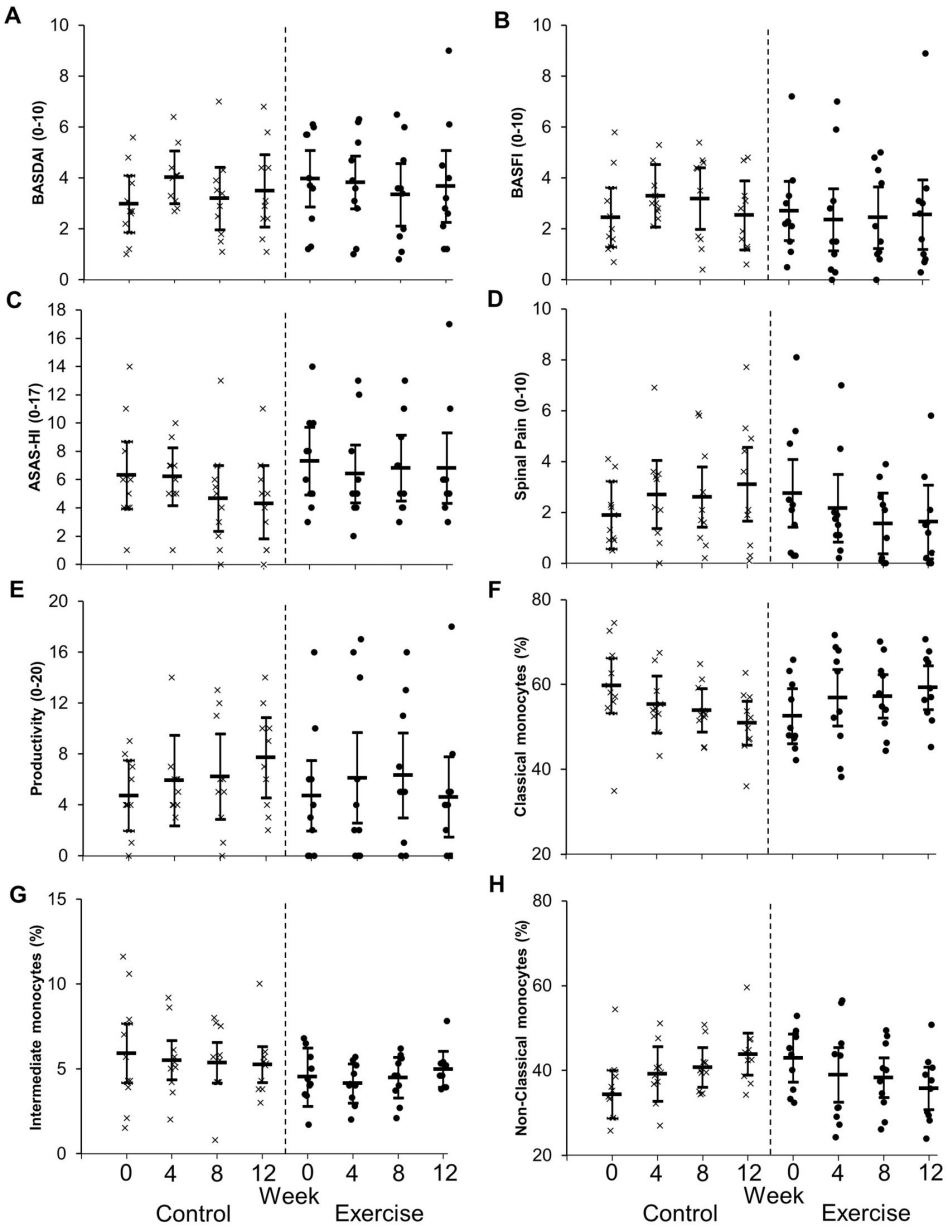
Subjective measures of symptom severity and proportions of immune cells in people with axSpA taking standard NSAID therapy who were randomly assigned into a control (*n* = 10) or exercise (*n* = 10) group at baseline (week 0). Control consisted of usual care. The exercise group walked for 30 min, five times per week, at a pace of somewhat hard. Statistical findings are presented in the text. Higher scores for BASDAI, BASFI, ASAS-HI, spinal pain and productivity indicate worse symptom severity

Spinal pain score decreased by 1.1 (95% CI −2.7, 0.5) in the exercise group and increased by 1.2 (95% CI −0.4, 2.9) in the control group (*P* = 0.049) (Fig. 3). There was a significant interaction for proportions and numbers of classical and non-classical monocytes (*P* ≤ 0.006). Proportions of classical monocytes increased in the exercise group by 6.7% (95% CI 1.0, 12.3) and decreased in the control group by 8.8% (95% CI −14.5, −3.1). The number of classical monocytes increased in the exercise group by 43 cells/µL (95% CI 1, 82) and decreased by 46 cells/µL in the control group (95% CI −87, −5). Proportions of non-classical monocytes decreased in the exercise group by 7.1% (95% CI −12.0, −2.2) and increased in the control group by 9.5% (95% CI 4.5, 14.4). The number of non-classical monocytes decreased in the exercise group by 33 cells/µL (95% CI −60, −6) and increased by 39 cells/µL in the control group (95% CI 12, 66). This coincided with a significant interaction for concentrations of IL-6 (*P* = 0.002) (Table 2). Concentrations decreased in the exercise group by 0.73 pg/ml (95% CI −1.07, −0.39) and increased in the control group by 0.10 pg/ml (95% CI −0.44, 0.24).

### Sensitivity analyses for CRP concentrations

**Figure 4.**
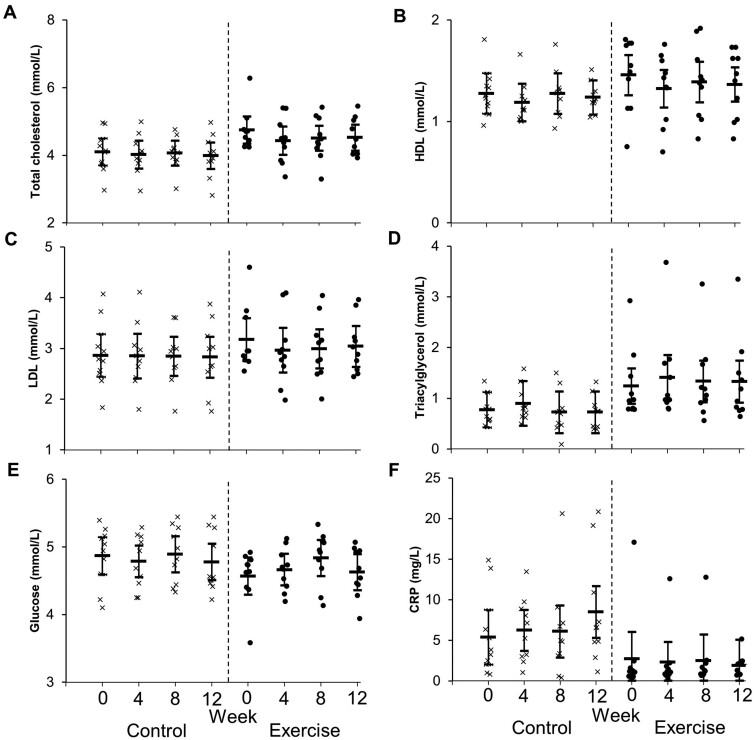
Concentrations of cardiovascular markers in people with axSpA taking standard NSAID therapy who were randomly assigned into a control (*n* = 10) or exercise (*n* = 10) group at baseline (week 0). Control consisted of usual care. The exercise group walked for 30 min, five times per week, at a pace of somewhat hard. Statistical findings are presented in the text. Data presented for all participants. Sensitivity analyses were conducted by removing outliers and repeating the statistical analyses (presented in the text). CRP: C-reactive protein; HDL: high-density lipoprotein cholesterol; LDL: low-density lipoprotein cholesterol

One participant in the exercise group had a baseline CRP concentration of 17 mg/L, which exceeded ±2.5 s.d. (Fig. 4). After removing the outlier, baseline CRP concentrations were significantly higher (*P* = 0.022, ES = 1.19) in the control group [5.36 mg/L (95% CI 2.90, 7.84)] than the exercise group [1.07 mg/l (95% CI 0.00, 3.67)]. Removing outliers did not change the interpretation for between-group trial × time interactions (*P* = 0.182).

### Sensitivity analyses for baseline differences

Adjustment for significant baseline differences in total cholesterol, CRP and non-classical monocytes between groups did not change the interpretation for any of these outcomes.

### 2 × 4 mixed ANOVA to assess whether any change in a key outcome was a result of an interaction between group and time

There were significant group × time interactions for proportions of classical and non-classical monocytes (*P* ≤ 0.012) but not for any of the other markers (*P* ≥ 0.080; Figs 3 and 4). Following up the classical monocyte interaction revealed proportions significantly increased in the exercise group at week 12 compared with week 0, but there were no significant effects at week 4 or 8 (Fig. 3). Classical monocytes significantly decreased in the control group at week 12 compared with week 0, but there was no significant effect at week 4 or 8 (Fig. 3). The proportion of non-classical monocytes was significantly lower at week 12 compared with week 0 for the exercise group, with no significant effect at weeks 4 or 8. The control group had significantly higher proportions of non-classical monocytes at weeks 8 and 12 compared with week 0, with no significant effect at week 4 (Fig. 3).

## Discussion

In line with the hypothesis, the results indicate that home-based walking for 12 weeks lowered the proportion and number of pro-inflammatory non-classical monocytes in people with axSpA due to an apparent phenotypic switch in monocyte profiles and an increase in classical monocyte proportions and numbers. Non-classical monocytes increased in the control group, with a decrease in classical monocytes. These changes coincided with lower IL-6 in the exercise group and higher concentrations in the control group. Further, spinal pain and systolic BP were lower in the exercise group and higher in the control group after 12 weeks. The data also suggest that walking exercise can reduce waist circumference and body fat percentage over 12 weeks, which are contributors to inflammation.

The lower proportion of non-classical monocytes with exercise is promising, as these are elevated in populations with inflammatory diseases [13]. The lower proportion reflects either different cells being released from the bone marrow or a switch within existing cell types. Non-classical monocytes secrete pro-inflammatory cytokines (TNF-α and IL-6), have high expression of toll-like receptor 4 and adhesion molecules (CCR5 and CX_3_CR1) and exhibit reactive oxygen species production, which contribute to inflammation and cardiovascular dysfunction [26, 27].

In the case of axSpA, the immune system mistakenly attacks healthy cells and in the process produces pro-inflammatory cytokines contributing to a state of chronic low-grade inflammation that is associated with heightened disease severity [28] and risk of CVD [29]. Accordingly, NSAIDs aim to suppress the action of inflammatory cytokines [28]. When ineffective, patients are progressed onto stronger biologic and novel targeted medications, which block receptors for ILs, Janus kinase and TNF-α and lower immune activity [6], but these are expensive and there is some evidence that certain biologic medications can increase the risk of infection due to a suppressed immune system [30].

Consequently, exercise may be an adjuvant method for improving cardiometabolic health and symptom burden with inflammatory diseases [31]. Cells of the immune system are key to inflammatory responses, but are also highly responsive to exercise and undergo numeric and phenotypic changes [32]. Exercise is known to encourage anti-inflammatory phenotype switching in monocytes, reducing the proportion of intermediate and non-classical monocytes [31]. This reduces pro-inflammatory cytokine production, down-regulating inflammatory pathways and lowering the risk of inflammatory diseases [31]. However, physical activity guidance for people with axSpA is vague. Individualized, structured aerobic exercise is listed without any direction towards exercise type, frequency, intensity or acknowledgement of physical limitations in an individual’s environment that may prevent exercise participation [6].

The present findings provide evidence that walking exercise can be used as an adjuvant therapy for people with axSpA on NSAID medication. The World Health Organization physical activity guidelines recommend 150 min of moderate-intensity activity per week for adults [33]. In the present study, people with axSpA tolerated 30 min of walking five times per week, as there was a 0% dropout rate in the study. To further support the use of walking in people with axSpA, the lowered proportion of non-classical monocytes with exercise coincided with lower CRP, IL-6 and systolic BP in the exercise group, whereas the control group had an opposite response. Although the control group did have non-significantly higher CRP and IL-6 concentrations at baseline, adjustment for differences did not change the interpretation of the findings. A recent (2022) review investigating the effect of exercise interventions in people with axSpA highlighted that aerobic exercise is effective in improving BASDAI and BASFI scores, as well as pain and mobility, even in the absence of significant changes in concentrations of inflammatory markers [8]. However, only 10 studies assessed markers of inflammation and 9 of these only measured CRP concentrations [8].

CRP is an acute-phase protein and is often used in clinical practice as a marker of systemic inflammation [15]. However, CRP may not fully represent the inflammatory process in axSpA due to low sensitivity and specificity and high variability, meaning large sample sizes are often needed to detect changes over time [15]. Further, CRP is only elevated in ≈40% of those living with axSpA and does not correlate with disease activity in >50% of those living with axSpA [15]. Consequently, only one previous study has documented significant changes in CRP concentrations with an exercise intervention, but the significant interaction was caused by stable CRP concentrations in the exercise group and a doubling of concentrations in the control group [34]. The present findings documented a similar trend, although the *P*-value was borderline significant (*P* = 0.092).

The present study advances understanding by showing concentrations of inflammatory markers improve with exercise, providing additional benefits to people with axSpA already on NSAIDs. Non-classical monocytes and IL-6 are indicative of an inflammatory environment and known contributors to mechanical stress and hypertension [35]. In the present study, non-classical monocytes and IL-6 were reduced with exercise, which coincided with a reduction in systolic BP and spinal pain, suggesting exercise can have anti-inflammatory effects and improve clinical measures such as systolic BP and spinal pain.

Loss of body fat is known to reduce the proportion of non-classical monocytes [36] due to less local tissue inflammation spilling over to other areas [37]. In the present study, body fat was 2.2% lower in the exercise group at follow-up but remained stable in the control group. However, adjustment for body fat percentage demonstrated negligible contributions to lower non-classical monocytes (*P*-value remained <0.001) and IL-6 (adjusted *P*-value of 0.004 compared with unadjusted *P*-value of 0.002). At baseline, there were no significant differences in the levels of physical activity between the groups. Therefore, it seems exercise lowers markers of inflammation independent of loss of body fat. IL-6 and non-classical monocytes increased in the control group even though body fat percentage remained stable, suggesting sedentary time may also independently increase inflammation in people with axSpA, but no studies have investigated the effects of breaking up sedentary time in people with axSpA.

Although BASDAI, BASFI and ASAS-HI scores decreased in the exercise group, indicating lower symptom severity, they were not significant. Typically, ≈80% of studies have reported significant BASDAI and BASFI improvements with exercise; however, exercise interventions are varied in type, duration, frequency and intensity [8]. Regarding the present study, subjective measures of symptom severity were all relatively low at baseline, which makes it difficult to see improvements with exercise. However, spinal pain did improve in the exercise group and became more problematic in the control group. Spinal pain is one of the major symptoms of axSpA and impacts mental health, engagement in physical activity and work productivity, as it immobilizes people with axSpA [38]. Therefore, it would be interesting to repeat the present study in people with axSpA who have had high-enough disease activity to necessitate the prescription of biologic or synthetic DMARD treatments. It may be the case that exercise has negligible effects on disease activity in those on biologic and/or synthetic DMARD treatments if medications are working. However, there may be additional benefits with increasing physical activity levels, such as improvements in mental health and maintaining muscle mass.

It is possible that the walking exercise in the present study created a positive cycle whereby chronic inflammation was reduced, lowering spinal pain, which then allowed people with axSpA to walk further, reducing inflammation. Future studies should assess the intervention for a longer duration with follow-up measures of habitual activity to see if this is the case. Nevertheless, STS5 and STS60 scores improved in the exercise group, suggesting higher physical function at follow-up, but the scores also improved in the control group, meaning improvements may be due to learned behaviour. Future studies should take this into consideration.

An arbitrary sample size was used due to a lack of existing monocyte data in people with axSpA, and this is the first study of this hypothesis. While a larger sample size may have provided sufficient power to detect significant differences in CRP concentrations and other markers, moderate–strong ESs were seen here, suggesting that some measures may have meaningful changes that are statistically non-significant. The small sample size is a limitation and larger sample sizes may be needed to detect statistically significant changes.

To conclude, this study suggests that supplementing NSAID therapy with walking exercise can improve inflammatory immune profiles in people with axSpA, which coincides with reductions in spinal pain. Importantly, the exercise was well tolerated, suggesting walking exercise can be used as an adjuvant anti-inflammatory therapy for NSAID treatments. This should now be explored in people living with axSpA with higher disease activity on prescribed biologic therapies.

## Supplementary Material

rkae062_Supplementary_Data

## Data Availability

Data will be made available on ClinicalTrials.gov after acceptance.
