# Supplementary material for: Exercise as an anti-inflammatory Therapy in Axial Spondyloarthritis Therapeutic Intervention (EXTASI) study: a randomized controlled trial
Source: Rheumatol Adv Pract. 2024 May 11;8(2):rkae062. doi: 10.1093/rap/rkae062 (PMC11157140; doi:10.1093/rap/rkae062)
Supplement: rkae062_Supplementary_Data [file rkae062_supplementary_data.docx]

**
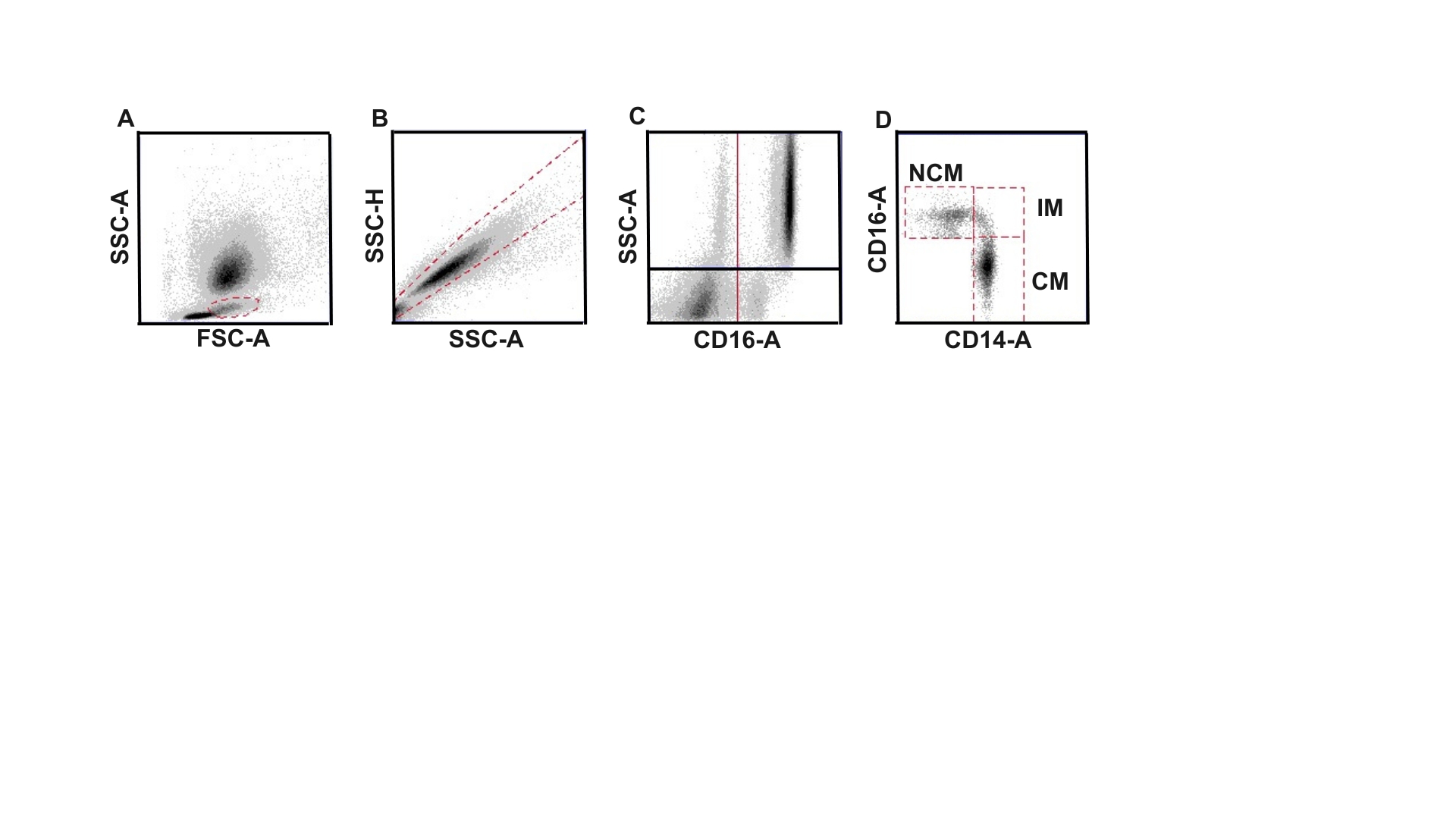
**

**Supplementary Figure S1.** Flow cytometry panel. A – monocytes were gated on using FSC-A vs SSC-A. B – doublets were excluded using SSC-A vs SSC-H. C – neutrophils were exluded using CD16-A vs SSC-A. Monocyte subsets were acquired by gating on monocytes, excluding doublets and neutrophils, then staining for CD14-FITC and CD16-PE. Compensation and positive regions were set using single stained controls. CM, classical monocytes; FSC-A, forward scatter area; IM, intermediate monocytes; NCM, non-classical monocytes; SSC-A, side scatter area; SSC-H, side scatter height.
